# HIV-related stigma and discrimination among health care workers during early program decentralization in rural district Gunungkidul, Indonesia: a cross-sectional study

**DOI:** 10.1186/s12913-022-07751-7

**Published:** 2022-03-17

**Authors:** Gaby G. Langi, Arie Rahadi, Ignatius Praptoraharjo, Riris A. Ahmad

**Affiliations:** 1 University Center of Excellence, AIDS Research Center, Health Policy and Social Innovation, Atma Jaya Catholic University of Indonesia, DKI Jakarta, 12930 Indonesia; 2 Field Epidemiology Training Program (FETP), Department of Biostatistics Epidemiology and Population Health, Faculty of Medicine Public Health and Nursing, Universitas Gadjah Mada, Yogyakarta, 55281 Indonesia; 3 Center for Health Policy and Management, Faculty of Medicine Public Health and Nursing, Universitas Gadjah Mada, Yogyakarta, 55281 Indonesia; 4 Center for Tropical Medicine, Faculty of Medicine Public Health and Nursing, Universitas Gadjah Mada, Yogyakarta, 55281 Indonesia

**Keywords:** Social stigma, HIV infections, Acquired immunodeficiency syndrome, Health facilities, Primary care, Decentralization, Task-shifting, Indonesia

## Abstract

**Background:**

Expanding HIV services by decentralizing provision to primary care raises a possible concern of HIV-related stigma and discrimination (SAD) from health care workers (HCWs) as new service points gain experience in HIV care delivery during early implementation. We surveyed indicators and examined the correlates of HIV-related SAD among HCWs in a decentralizing district of rural Gunungkidul, Indonesia.

**Methods:**

We conducted a cross-sectional survey on a random stratified sample of 234 HCWs in 14 public health facilities (one district hospital, 13 primary health centers [PHC]) during the second year of decentralization roll-out in the district. We computed the prevalence of SAD indicators and used multivariable logistic regression to identify the correlates of these SAD indicators.

**Results:**

The prevalence of SAD among HCWs was similarly high between hospital and PHC HCWs for fear of HIV transmission (~71%) and perceived negative image of PHIV (~75%). Hospital HCWs exhibited somewhat lower avoidance of service duties (52.6% *vs*. 63.7%; *p* = 0.088) with weak evidence of a difference and significantly higher levels of discriminatory practice (96.1% *vs*. 85.6%; *p* = 0.009) than those working in PHCs. Recent interactions with PLHIV and receipt of training lowered the odds of fear of HIV transmission (*p* <0.021). However, the odds of avoiding care duties increased with receipt of training (*p* =0.003) and decreased for hospital HCWs (*p* = 0.030). HIV knowledge lowered the odds of discriminatory practice (*p* = 0.002), but hospital facility and nurse/midwife cadres were associated with increased odds of discriminatory practices (*p* <0.021). No significant correlate was found for perceived negative image of PLHIV.

**Conclusion:**

HIV-related SAD among HCWs can be prevalent during early decentralization, highlighting the need for timely or preparatory interventions with a focus on building the capacity of hospital and non-physician workforce for positive patient-provider interactions when delivering HIV care.

**Supplementary Information:**

The online version contains supplementary material available at 10.1186/s12913-022-07751-7.

## Background

Stigma and discrimination (SAD) towards people living with HIV (PLHIV) remains widespread across the globe and particularly in low- and middle-income countries (LMICs) [1–3]. More than half of adult residents sampled in population surveys on SAD reported at least one HIV-related SAD attitude within their lifetime [4] and in every PLHIV experienced a denial of health service since their diagnosis [5]. The advent of antiretroviral treatment (ART) has made HIV-related SAD increasingly less associated with debilitating disability or illness resulting from immunocompromised health [6, 7], but more embedded in a multitude of behavioural and social identities that characterize the HIV key populations as possible targets of SAD treatment [8]. In this sense, SAD is a product of complex interactions of structural impetus (e.g., discriminatory policies, societal norms, economic opportunities for marginalized groups) and interpersonal factors (e.g., levels of social support, the prevailing attitude in the household or at the organizational level) with individual characteristics (e.g., educational attainment, employment, sexual orientation, substance abuse) [9–11], which collectively augments the normative distinctions of the HIV key populations from the rest of the masses. Consequently, the types of SAD, its sources or perpetrators, and the mechanism of action in relation to health outcomes for each type are governed by the patterns of interaction within and between these determinants at various population levels [12].

Much of the early theoretical ground to distinctively capture and measure SAD in numerical indices was derived from the work of Goffman (1963) who laid the foundation of what constitutes stigma in terms of perpetrated actions (to devaluate) and the impact these inflicted on others (the feeling of discredit) [13]. The conceptualization by Link and Phelan (2001) describes the cognitive and attitudinal process of stigma by active labeling of differences and stereotyping and by separating and eventually excluding those stigmatized from a relevant social context, thereby equating stigma with discrimination [14]. More recent theoretical work clarifies the cognitive process of SAD in which stereotyped ideas or imagined contra attitudes condition future discriminatory actions enacted as a response [15, 16]. Within this framework, SAD is underpinned by principal human faculties that manifest in knowledge or its lack thereof (ignorance), attitude (prejudice), and actions (discrimination) [17]. Once applied on target victims, SAD works to compel PLHIV or those associated with the infection to modify their health behaviours and current access to care in response to enacted service barriers (enacted stigma), devalued self-worth from internalizing unfavourable societal views (internalized stigma), and revised expectations of foreseeable negative treatment from others (anticipated stigma) [12]. SAD therefore can be broadly viewed as a continuum of process [18] comprising two distinct but related endpoints that accrue to the presumed perpetrators, for whom attitudinal indicators or discriminatory practices are its final outcomes, or to the victims, for whom health detriments or declining health status are its final outcomes.

In health care delivery, the setting of the current study, health care workers (HCWs) are in position of power over entry into care and its continual adjustments during chronic treatment to maximize health outcomes while also serving in the best interest of the patient [19]. Evidence demonstrates that SAD in health care setting restricts access to HIV testing and diagnosis, disincentivizes uptakes of antiretroviral treatment (ART), erodes treatment adherence, and compromises the quality of life of PLHIV [20–24]. The deleterious consequences of suboptimal health behaviors in morbidity and mortality [25] place health care delivery in a prominent rank among the priority sectors to target for SAD elimination [26].

Although experiences of SAD by PLHIV in the health care setting are widely documented [27–29], examination of SAD attitudes and practices among health care workers (HCWs) in the context of decentralized care has received less attention. The expansion of HIV care and treatment in LMICs over the last two decades brought about innovations to redistributing service capacity more evenly in the health system in response to increased demand by decentralizing provision down to primary care and across the ranks of health care cadres [30]. Advances in diagnostic and antiretroviral technologies have simplified and standardized care procedures to the point of becoming amenable to decentralized provision by non-physician clinicians at the primary care level [31]. HIV care decentralization provides a unique programatic context for emergent SAD in which a wider scope of HCWs is introduced first-hand to the care engagement of PLHIV and key populations.

Investigations into decentralized programming indicate fear of discrimination and unwarranted disclosure of HIV status as two areas of SAD that PLHIV feel strongly concerned about or commonly experienced during health care delivery [32, 33]. Firstly, these concerns may signal an early progress in organizational learning as primary health centres (PHCs), now an emerging model of HIV care provision in the health system, rearrange resource allocations in adapting to the imperative of HIV service provision. This contrasts with the referent hospital whose pioneering HIV care in the community has reached maturity. Gaps in supply-side determinants of quality of care such as differential capacity building, skills composition, experience in service provision, and other resource endowments between types of facility units in a decentralization setting can therefore inform priority areas of improvement when transitioning to a decentralized model of HIV care. Secondly, in maturing decentralization programs and rural areas the same concerns may also foretell a possible trade-off for a segment of patients who changed or maintained their access location at a PHC close to their residence when the cost of obtaining better quality of care at the referent hospital exceeds that of access convenience [34–36]. Seen from this perspective, the predominant type of SAD in HCWs and its effect on PLHIV patients can differ by the stage of program maturity or geographical context in which decentralized HIV care is instituted. For instance, actioned discrimination such as denial of services by HIV serostatus or membership to a key population may be more prevalent during early decentralization as the human resource capacity is being built to target these actions [35], whereas a vestige of attitudinal SAD and stigmatizing beliefs may persist in a later stage of program cycle. The indication of differential quality of care receives little attention in comparative effectiveness reviews of HIV care decentralization programs in LMICs, which have concluded non-inferior outcomes of PHCs or other equivalently decentralized provision relative to the pioneering hospital [30, 37–39]. The summary effect sizes reported in these reviews mask the impact of various stages of program maturity in the included studies. Similarly, existing studies on HIV-related SAD among HCWs are limited in the scope of health care professions or had a focus on mature programs [40–43], which forego the dynamics in patient-provider interactions and emergent SAD during early decentralization.

The global commitment to HIV-related SAD has been recognized on an equal footing with efforts to eliminate new HIV infections and AIDS-related deaths [44]. In 2018, UNAIDS launched a partnership to support countries in protecting the human rights of PLHIV and advance country capacity to reduce SAD [26]. Within the short timespan since the launch, changes to the baseline levels of SAD have been reported in varying degrees, with most countries failing to achieve the elimination target by the end of 2020 [27]. Recent data reveal that the proportions of PLHIV experiencing SAD during health care delivery varied greatly in the range of 1.7% to 21.0% [27], which suggests different trajectories in the capacity to reduce SAD that may be specific to the health system in each locality [45].

The HIV epidemic in Indonesia is characterized by stable growth in annual incidence and a concentration of disease burden in a few key populations such as men who have sex with men (MSM), people who inject drugs (PWID), and sex workers [46]. ART is provided free at the point of use, but coverage which currently stands at 26% of an estimated 540,000 PLHIV is among the lowest in the region [46] and the true rates of community viral suppression remain largely undocumented due to routine viral load monitoring being financed out-of-pocket. In 2012 the Ministry of Health launched a nationwide campaign to surge the diagnostic and treatment capacity in the wake of new projections of HIV cases, targeting phased service expansion to existing PHCs and peripheral facilities in anticipation of a growing demand for immediate ART for key populations and other priority groups such as HIV-infected pregnant women and tuberculosis coinfected patients [47]. To date, there are 1.951 health care facilities in the country with the capacity in ART initiation and management in addition to HIV diagnosis, of which 61% are PHCs [48].

A growing body of research on HIV-related SAD in the country has documented the health burden of SAD in PLHIV in relation to its negative effect on adherence to ART in urban and rural districts [49, 50]. Two studies examined beliefs and attitudinal HIV-related SAD in HCWs, specifically among nurses and in multiple HCW cadres practicing in a low caseload setting [42, 51]. While these studies document high SAD in PLHIV with average value measures consistently exceeding half the plausible range, neither compared between types of health care facility and nor was situated in rural areas where care decentralization is most relevant to overcome distance-related barriers to health care access [34–36]. We also note the dearth of international studies measuring HIV-related SAD in the setting of care decentralization, as we pointed above. Herewith we present findings from a survey of HCWs, describing the prevalence and examining the correlates of attitudinal and behavioural indicators of SAD against PLHIV during early decentralization in rural Gunungkidul, in the direction of recommending actions to tackle HIV-related SAD in this context. The negative effects of HIV-related SAD on sustained chronic HIV care [20–24, 49, 50, 52] runs counter to the benefit of decentralization in creating new capacity for treatment and promoting more equitable access in close proximity to the community PLHIV and their families reside, particularly in rural areas with a sparse distribution of health care providers.

## Methods

### Study design and setting

We conducted a cross-sectional survey in Gunungkidul, a rural district in Yogyakarta Province, with data collection from December 2016 to March 2017. The district has the lowest human development index in the province [53]. Two hospitals and 30 PHCs serviced an estimated 700.000 residents of the district in 2016 [54]. In 2016 there were 238 documented HIV cases with most diagnoses occurring in late clinical stages [55]. In response to the growing number of cases, the District Health Office decentralized provision of HIV testing and care from the district hospital to 13 PHCs beginning in 2015. The process of decentralizing HIV services began with developing PHCs capacity through trainings related to HIV testing and counselling, care, support and treatment (CST), prevention mother to child transmission (PMTCT), and HIV information system. The initiative expanded the role of PHCs to HIV testing, referrals of hospital-based ART initiation, management of stable ART patients, and treatment of non-severe opportunistic infections.

### Participants and sample size

We surveyed HCWs at the district referent public hospital and 13 PHCs participating in the decentralization program. HCWs were eligible to participate in the survey if they had >12 months of service with the current facility in a medical or non-medical area. Health care professions comprised physicians (specialists and general practitioners), dentists, registered nurses, midwives, and a class of health care cadres with limited or no medical duties, including nutritionists, physiotherapists, laboratory or radiology technicians, medical record officers, and public health experts. We powered the survey to detect 64% HIV-related SAD in a stratified sampling design [56] and recruited 234 HCWs, a response rate of 99% of the total 235 targeted. We stratified recruitment by facility type, allocated recruitment evenly between the number of hospital and PHC respondents, and randomly sampled HCWs in each stratum proportional to observed HCW size in each facility unit.

### Study instruments

The survey questionnaire on SAD indicators was adapted from *Measuring HIV Stigma and Discrimination among Health Facility Staff* prepared by The Health Policy Project [57]. The questionnaire also had an extended module on SAD in the context of prevention of mother-to-child HIV transmission. Data from this module were not utilized in this study. The main questionnaire was divided into five sections: one for demographic information including HIV knowledge and the remaining for various SAD indicators and in-facility HIV policy environment. The questionnaire has been used in diverse settings [41, 58] and proved simple to administer or for self-administration without much overhead in duration or cognitive effort. In the study setting, we piloted the translated questionnaire in 30 HCWs from the hospital prior to use in survey respondents, which demonstrated satisfactory inter-item reliability (Cronbach’s alpha >0.70 for all sections). Selected HCWs were contacted to participate in the survey. Interested HCWs then received hardcopy questionnaires, an informed consent form, and survey instructions via courier or delivered in-person by a team member for those who wished to complete the questionnaire on the same day. We allowed a one-week period during which HCWs were expected to complete the questionnaire, and added a maximum of another one week with 2-3 times follow up phone calls for those who did not complete the questionnaire. Respondents self-completed the questionnaire in 20-35 minutes.

### Study outcomes and other variables

Four SAD indicators were constructed from responses in the two sections of the questionnaire eliciting personal opinions on HIV infection control and PLHIV and key populations. SAD indicator ‘fear of HIV transmission’ pertains to levels of worry (from ‘not worried’ to ‘very worried’) when performing medical duties with PLHIV patients involving direct contact with the clothing, dressing the wounds, blood drawing, and temperature check (Cronbach’s alpha = 0.86). ‘Perceived negative image of PLHIV’ corresponds to levels of agreement (from ‘strongly agree’ to ‘strongly disagree’) with exemplary statements on their perceived disregard for infecting others, presumptive promiscuity, reckless risk behaviors, and whether PLHIV deserve shame and HIV is believed to be a punishment for their risky behaviors (Cronbach’s alpha = 0.70). The third SAD indicator was assessed as how respondents would approve (from ‘strongly agree’ to ‘strongly disagree’) ‘avoidance of service duties’ for MSM, PWID, and sex workers, the HIV key populations, if given the opportunity (Cronbach’s alpha = 0.88). Lastly, ‘discriminatory practices’ refer to unjust or excessive precautions (‘yes’ and ‘no’) in contact avoidance, double gloving, being gloved up throughout the entire care episode, or use of special infection-control measures that HCWs would not apply when caring for non-HIV patients (Kuder-Richardson 20 = 0.92). All SAD indicators used a four-item Likert scale except for discriminatory practices. HCW cadres with core duties not relevant to one or more task described in a question set for a SAD indicator could select a ‘not applicable’ option. Fear of HIV infection and discriminatory practices were specific to medical cadres in direct health care delivery. All respondents provided responses to the remaining SAD indicators.

We included age, sex, education (< and >bachelor’s degree), HIV knowledge (scale: 0—10), facility type (hospital and PHCs), HCW cadres (physician, nurse, other professions), interactions with PLHIV, and receipt of training in HIV and SAD topics. HIV knowledge test comprised standard 10 questions on basic knowledge of HIV transmission and its mode of exposure [59, 60], with a total score accumulated on correct answers.

### Statistical analysis

We described sample characteristics in mean and standard deviation for continuous variables and counts and proportion for binary or categorical variables, stratified by facility type. We formed dummy indicators of SAD by dichotomizing all Likert-like responses at the mid category, which are ‘worried’ and ‘very worried’ for fear of HIV infection and ‘agree’ and ‘strongly agree’ for both perceived negative image and avoidance of service duties. Discriminatory practices were present if the respondent reported any unnecessary preventive measure. Prevalence of SAD indicators was then computed in a similar manner. Differences by facility type were evaluated using the Student’s t-test or Pearson’s chi-squared test. As we were concerned that our broad stratification may lead to imbalanced proportions of HCWs sampled across PHCs, we evaluated the effect of these differential sampling rates on SAD prevalence using binomial regression with the logit link function for fractional, prevalence outcomes in the 0—1 range [61]. Table A1 in the supplement to this article reports no evidence of association between PHC sampling rates and the prevalence estimates on all SAD indicators (*p* >0.308). Multivariable logistic regression was used to explore correlates of SAD indicators. We removed education from the final model as this variable was deemed redundant in differentiating groups of HCW cadres between physicians, who all had at least a bachelor’s qualification, and others. Sampling weight adjustments were applied to adjust for non-response from hospital facility and discrepant distribution by strata of facility type. All *p*-values <0.050 were considered to provide sufficient evidence of statistical significance. Stata version 14.2 (College Station, TX) was used for all analyses.

## Results

### Characteristics of respondents

A total of 234 HCWs participated in the survey with an equal proportion of HCWs from the hospital (n = 116) and PHCs (n = 118). HCWs were on average 40 years old at the time of survey with a female majority, and could answer correctly eight questions on basic knowledge of HIV transmission and its mode of exposure (Table 1). PHC HCWs tended to be more highly educated with over 40% having at least a bachelor’s qualification compared to approximately 30% of their hospital counterparts. A majority were in the nursing or midwifery profession with slightly more physicians and other professions working at PHCs. Significantly more hospital HCWs (63.8%) than PHC HCWs (44.9%) had recent interactions with PLHIV (*p* = 0.004). Most HCWs had yet to receive any training in HIV and SAD, infection control and universal precautions, informed consent and patient confidentiality, or SAD in HIV key populations. All characteristics except HIV knowledge and recent interactions with PLHIV in 12 months were broadly similar for HCWs from either facility type.

**Table 1:** Respondent characteristics by facility type

	Total	Facility type		
Characteristic	*Mean (SD)*	*Mean (SD) or n (%)*		***p-***value^a^
	*or n (%)*	Hospital	PHC	
	(*n* = 234)	(*n* = 116)	(*n*= 118)	
Age (years)	40.1 (8.2)	39.4 (8.6)	40.9 (7.60)	0.193
Sex				0.546
Female	157 (67.1%)	80 (68.8%)	77 (65.2%)	
Male	77 (32.9%)	36 (31.3%)	41 (34.8%)	
HIV knowledge (scale:1—10)^b^	8.1 (1.3)	7.9 (1.5)	8.3 (1.0)	0.009
Education				0.092
<Bachelor degree	151 (65.4%)	81 (69.8%)	70 (59.3%)	
>Bachelor degree	83 (35.5%)	35 (30.2%)	48 (40.7%)	
Profession				0.988
Physician/dentist	35 (15.0%)	14 (12.1%)	21 (17.8%)	
Nurse/midwife	148 (63.2%)	80 (69.0%)	68 (57.6%)	
Other	51 (21.8%)	22 (18.9%)	29 (24.6%)	
Interactions with PLHIV in 12 months				0.004
No	107 (45.7%)	42 (36.2%)	65 (55.1%)	
Yes	127 (54.3%)	74 (63.8%)	52 (44.9%)	
Receipt of training, by topic				
HIV and SAD				0.127
No	195 (83.3%)	94 (79.7%)	101 (87.1%)	
Yes	39 (16. 7%)	24 (20.3%)	15 (12.9%)	
Infection control & precautions				0.075
No	160 (68.4%)	73 (62. 9%)	87 (73.7%)	
Yes	74 (31.6%)	43 (37.1%)	31 (26.3%)	
Informed consent & confidentiality				0.694
No	188 (80.3%)	92 (79.3%)	96 (81.4%)	
Yes	46 (19.7%)	24 (20.7%)	22 (18.6%)	
SAD in HIV key populations^c^				0.259
No	204 (87.2%)	104 (89.7%)	100 (84.7%)	
Yes	30 (12.8%)	12 (10.3%)	18 (15.3%)	
Any topic				0.118
No	145 (62.0%)	67 (57.8%)	78 (66.1%)	
Yes	89 (38.0%)	49 (42.2%)	40 (33.9%)	

*HIV* human immunodeficiency virus; *PHC* primary health center; *PLHIV* people living with HIV; *SAD* stigma and discrimination; *SD* standard deviation

^a^For the difference between hospital and PHC health care workers using the Student’s t-test for continuous variables and the Pearson's chi-squared test for binary and categorical variables

^b^Scores correspond to the number of correct answers out of 10 questions on basic knowledge of HIV transmission and its mode of exposure. The breakdown for each item and its proportion of correct response (in brackets) is as follows: HIV can be prevented by: 1) being faithful to one's husband or wife (65.8%); 2) wearing condoms during sex with persons of unknown HIV status (89.3%); 3) ref-raining from sharing needles (93.6%); 4) eating from the same plate that PLHIV used (91.9%); 5) mosquito bites (88.5%); 6) the risk of getting infected from contaminated needles is 1:300 (32.5%); 6) mother’s milk can be a vehicle for HIV transmission (65.0%); 8) sperm or semen can be a vehicle for HIV transmission (91.9%); 9) vaginal discharge can be a vehicle for HIV transmission (95.7%); and 10) other bodily fluids containing blood can be a vehicle for HIV transmission (95.7%)

^c^These are people who inject drugs, men who have sex with men, and sex workers

### Prevalence of HIV-related SAD

**Fig. 1.**
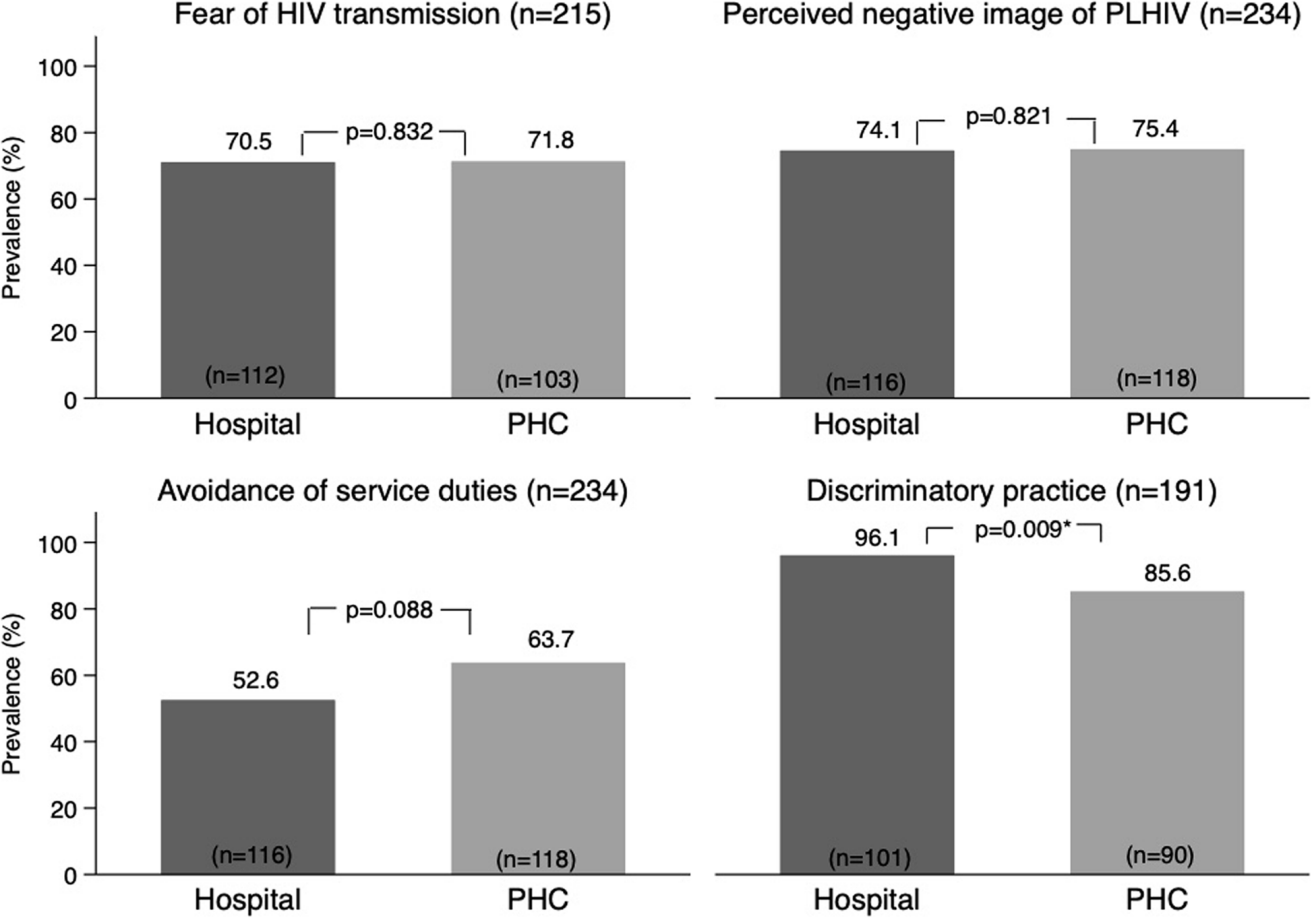
Prevalence of SAD indicators by facility type. HIV = human immunodeficiency virus; PHC = primary health center; PLHIV = people living with HIV; SAD = stigma and discrimination. Fear of HIV transmission: how worry staff are with the prospect of HIV transmission when caring for PLHIV; perceived negative image of PLHIV: unfounded beliefs, presumptions of negative behaviors of PLHIV; avoidance of service duties: omission, neglect to provide services for HIV key populations if such an option becomes feasible; discriminatory practice: unnecessary preventive measures taken when caring for PLHIV

Figure 1 presents the prevalence estimates of perceived HIV-related SAD. Perceived SAD was prevalent, in rates greater than 50%, in all four indicators for both facility types. Of 215 HCWs who were eligible to provide responses, approximately 71% in both facility types feared contracting HIV transmission when caring for PLHIV patients. There was a similarly high prevalence of hospital and PHC HCWs who perceived a negative image of PLHIV (~75%) out of all HCWs. Close to 64% of all PHC HCWs would avoid service duties for HIV key populations if and when it became feasible to do so. The prevalence of this indicator was lower for hospital HCWs (~53%), and the difference trended towards significance (*p* = 0.088). Discriminatory practices were the most prevalent SAD indicator, reported by over 80% of 191 HCWs involved in health care delivery, and were significantly higher for hospital HCWs than their PHC counterparts (~96% vs. ~85%; *p* = 0.009).

### Correlates of HIV-related SAD

Table 2 presents the correlates of HIV-related SAD among HCWs in all four SAD indicators from multivariable analyses. The odds of fear of HIV transmission were approximately half as much for HCWs who had recent interactions with PLHIV (adjusted odds ratio [aOR] = 0.44; 95% confidence interval [CI] = 0.22—0.87; *p* = 0.017) and received any training on HIV and SAD (aOR = 0.47; CI = 0.25—0.89; *p* =0.021). No correlate was found for perceived negative image of PLIV as there was no significant association between any of the characteristics and this SAD indicator. Hospital HCWs appeared less likely to avoid service duties for HIV key population compared to PHC HCWs (aOR = 0.53; CI = 0.30-0.94; *p* = 0.030). Receipt of training increased the odds of avoiding service duties for HIV key populations by more than two-fold (aOR = 2.46; CI = 1.37—4.42; *p* = 0.003). The odds of discriminatory practice lowered with the increase of HIV knowledge (aOR = 0.23; CI = 0.09—0.60; *p* = 0.002), and increased for hospital HCWs (aOR = 4.42; CI = 1.24—14.34; *p* = 0.021) or non-physician cadres, including nurses/midwives (aOR = 6.21; CI = 1.55—24.88; *p* = 0.010).

**Table 2:** Correlates of HIV-related stigma and discrimination

	Fear of HIV			Perceived negative			Avoidance of service			Discriminatory		
Characteristic	transmission^a^			Image of PLHIV^a^			Duties^a^			practice^a^		
	(*n* = 215)			(*n* = 234)			(*n* = 234)			(*n* = 191)		
	aOR	95% CI	*p*-value	aOR	95%CI	*p*-value	aOR	95%CI	*p*-value	aOR	95%CI	*p*-value
Age	1.02	0.98-1.06	0.320	1.01	0.97-1.05	0.548	0.99	0.95-1.02	0.493	1.09	0.99-1.21	0.078
Sex (male)	0.80	0.37-1.73	0.574	1.50	0.72-3.13	0.282	0.71	0.38-1.33	0.288	0.24	0.05-1.08	0.053
HIV knowledge^b^	1.04	0.82-1.33	0.715	1.00	0.77-1.29	0.995	0.95	0.77-1.16	0.596	0.23	0.09-0.60	0.002
Facility type (hospital)	1.11	0.59-2.14	0.732	0.95	0.50-1.81	0.897	0.53	0.30-0.94	0.030	4.42	1.24-14.34	0.021
Profession			0.121			0.437			0.599			0.034
Physician/dentist	Reference			Reference			Reference			Reference		
Nurse/midwife	2.24	0.96-5.24	0.061	1.58	0.68-3.63	0.284	1.02	0.48-2.17	0.953	6.21	1.55-24.88	0.010
Others^c^	1.34	0.49-3.65	0.565	1.93	0.67-5.52	0.218	1.44	0.59-3.54	0.423	3.95	0.65-23.62	0.133
Interactions with PLHIV	0.44	0.22-0.87	0.017	0.99	0.53-1.88	0.992	1.32	0.73-2.37	0.346	0.52	0.15-1.85	0.315
in 12 months (yes)												
Receipt of any training	0.47	0.25-0.89	0.021	0.87	0.46-1.64	0.670	2.46	1.37-4.42	0.003	0.71	0.22-2.31	0.570
(yes)^d^												

*aOR* adjusted odds ratio; *CI* confidence interval; *HIV* human immunodeficiency virus; *PLHIV* people living with HIV

^a^Fear of HIV transmission: how worry staff are with the prospect of HIV transmission when caring for PLHIV; perceived negative image of PLHIV: unfounded beliefs, presumptions of negative behaviors of PLHIV; avoidance of service duties: omission, neglect to provide services for HIV key populations if such an option becomes feasible; discriminatory practice: unnecessary preventive measures taken when caring for PLHIV

^b^Measured using 10 questions on basic knowledge of HIV transmission and its mode of exposure

^c^A class of cadres with limited or no medical duties, including nutritionists, physiotherapists, laboratory or radiology technicians, medical record officers and public health experts

^d^Training covers one or more of the following topics: HIV and stigma and discrimination; infection control and universal precautions; informed consent and patient confidentiality; and stigma and discrimination in HIV key populations

## Discussion

The prevalence of SAD during early decentralization was high as found in this setting and in equivalently concerning levels for all the four indicators despite some notable differences between hospital and PHC HCWs. Highly prevalent discriminatory practices, exceeding 90% and 80% of HCWs at the district hospital and PHCs respectively, may indicate a lack of understanding of infection control, as evidenced from the low training coverage. Different indicators of SAD seem to have unique correlates in the type and direction of effect. In general, systemic correlates encapsulating individual HCWs within their workplace or health care professions exert a greater likelihood of SAD than do demographic correlates or those related to competency such as training, HIV knowledge, and service interactions.

A rather unusual finding is related to how training can have an opposing influence on certain SAD indicators. In this case, training was associated with a reduction in the odds of fear of transmission and unexpectedly increased avoidance of service duties. Our liberal definition of training as any receipt in one or more competency topic may misrepresent the training effect in its association with service avoidance. We defined training as such since some of the training topics may broadly correspond to more than one SAD indicator. This approach could, however, underplay specific trainings that are more relevant to service avoidance and result in a statistical artifact for other SAD indicators that share little in construct with fear of HIV transmission. It is also possible that relevant training materials, particularly in infection control, missed the emphasis on due safety precautions or that protective facility-level policies such as access to post-exposure prophylaxis were minimal so that this condition exaggerated the nosocomial risk of HIV transmission to the point of avoiding care duties.

This study showed that the increase of HIV knowledge would reduce the odds of discriminatory practice. The association of HIV knowledge with discriminatory practices was in line with the results of prior studies [51, 62]. No other SAD indicators were correlated with HIV knowledge. Improving knowledge is essential and needs be recognized as one of the factors that condition or mediate SAD-preventive behaviours as found in other studies of HCWs or other populations in LMICs [63–65].

Discriminatory practices are more prevalent in the hospital and among the non-physcian cadres, especially nurses/midwives. HIV patients seeking care at the hospital tend to be in late clinical stages and have a worse prognosis, requiring more invasive procedures that may subject ill-informed HCWs to excessive prevention in the presence of a perceived elevated threat of HIV and opportunistic infections. As decentralization program matures, more burden of acute care will be alleviated through expanded health system capacity to diagnose and enroll a greater number of PLHIV into ART care at PHCs [34, 65, 66], and this growth in implementation can be expected to contribute to a reduction of excess SAD among hospital HCWs. SAD in non-physician cadres can be attributed to the physician-centered model of care preceding decentralization that placed nurses or other medical professions in support roles, with a limited functional scope in the delivery of vital health care for PLHIV such as management of opportunistic infections or ART prescribing. Decentralization taps into the supply of non-physician cadres and empowers them to assume clinical leadership in HIV care with documented success in other LMICs [37, 38]. Quasi-experimental evidence demonstrates SAD reduction among nurses after participation in health delivery leadership programs [67].

Given the high prevalence of SAD among HCWs in this early decentralization setting, capacity building activities to combat SAD can are needed. Among the top priorities is special trainings on HIV-related SAD with a hands-on ap proach to nurture effective and culturally competent service interactions that uphold the rights and dignity of PLHIV and HIV key populations. A review of the existing national curricula, which HCWs must complete to hold a professional certification in HIV care, can identify gaps in modules, program structures, and learning methodologies to better adapt to the needs of HIV key populations and to the demand of decentralized programs in clinical leadership roles for nurses and other relevant cadres, and to the prevailing communal culture in the district to strategically promote respect and autonomy over HIV status disclosure associated with occurrence of SAD [68]. Engagement with PLHIV groups as experts in the review process, content updating, and training facilitation will ensure that curricula stay abreast of emerging community perspectives.

Current approaches to capacity building allow piecemeal deliveries where a curriculum or a competency program is completed on standalone topics accumulated over a period of time. While offering flexibility, these approaches can delay completion of essential skills and result in partial competence. Systematizing training deliveries coherently for a comprehensive coverage of topics is another area of improvement in capacity building. Preferably, all essential trainings should be completed for all HCW cadres prior to or at the earliest time around the decentralization program roll-out. Additionally, staffing policies that reward staff retention is needed to facilitate selection of highly-motivated individuals into HIV care decentralization programs, promote specialization in HIV care, and balance against outflows of competent HCWs due to compulsory job rotation and transfers in public health [69].

Our findings should be interpreted with caution. Apart from the artifactual problem described above, the training effect on SAD can also be an outcome of self-selection where the trainings attracted participation from HCWs with persistent discriminatory attitudes or those who reasonably protect themselves from nosocomial HIV transmission rather than vice versa due to the cross-sectional design. Secondly, two SAD indicators depicted in this study, namely perceived negative image and avoidance of service duties, evaluate perceptions and hypothetical (in)actions which may or may not bear resemblance to the actual behavior of HCWs and therefore should not be construed as a definite form of enacted SAD. Thirdly, we do not feel that social desirability drove our results given the prevalent SAD found in the study and the self-administration of the survey, which minimized the likelihood of responses being falsely congruent with the expectations of the research team. Lastly, our survey participants encompassed a wider pool of health care cadres, with some non-medical professions having minimal exposure to HIV and thereby elevating the SAD prevalence as compared to what would be expected if participation was limited to medical professions.

Program implementation in high-SAD environment such as the study setting can benefit from the development and evaluation of innovations in capacity building of HCWs to reduce SAD. Tracking of SAD indicators over time can give insights into how SAD evolves through phases of program maturity and impacts on long-term patient outcomes.

## Conclusion

Early decentralization is a critical period with possible high SAD in service delivery as a broad spectrum of HCW cadres participate in rapidly expanding services to anticipate the surging demand for HIV care. Facility type in which HCWs provide services and types of HCW cadres, HIV knowledge and training, are the strongest correlates of SAD. Pre-decentralization preparatory work or timely interventions in capacity building, emphasizing professional, cultural, and practical competencies to create safe and emphatic interactions during health care delivery, can reduce SAD among HCWs going forward.

## Supplementary Information

**Additional file 1.**

## Data Availability

The datasets used and/or analysed during the current study are available from the corresponding author on reasonable request.

## Abbreviations

aOR: Adjusted odd ratio
ART: Antiretroviral treatment
CI: Confidence interval
HCWs: Health care workers
HIV: Human immunodeficiency virus
LMICs: Low-and middle-income countries
MSM: Men who have sex with men
PMTCT: Prevention mother to child transmission
PHCs: Primary health centers
PLHIV: People living with HIV
PWID: People who inject drugs
SAD: Stigma and discrimination

## References

[1] UNAIDS. Seizing the moment: tackling entrenched inequalities to end epidemics (Global AIDS update 2020). 2020. https://reliefweb.int/sites/reliefweb.int/files/resources/Global%20AIDS%20Update%202020%20-%20Seizing%20the%20moment%20-%20Tackling%20entrenched%20inequalities%20to%20end%20epidemics.pdf. Accessed 21 Mar 2021.

[2] Smith MK, Xu RH, Hunt SL, Wei C, Tucker JD, Tang W, Luo D, Xue H, Wang C, Yang L, Yang B (2020). Combating HIV stigma in low-and middle-income healthcare settings: a scoping review. Journal of the International AIDS Society..

[3] Kane JC, Elafros MA, Murray SM, Mitchell EM, Augustinavicius JL, Causevic S, Baral SD (2019). A scoping review of health-related stigma outcomes for high-burden diseases in low-and middle-income countries. BMC medicine..

[4] UNAIDS. On the fast-track to end AIDS by 2030: focus on location and population. 2015. https://www.unaids.org/en/resources/documents/2015/FocusLocationPopulation. Accessed 21 Mar 2021.

[5] UNAIDS. Make some noise for zero discrimination: zero discrimination day 1 march 2017. 2017. https://www.unaids.org/sites/default/files/media_asset/2017-zero-discrimination-day_en.pdf. Accessed 22 Mar 2021.

[6] Tsai AC, Bangsberg DR, Bwana M, Heberer JE, Frongillo EA, Muzoora C (2014). How does antiretroviral treatment attenuate the stigma of HIV? Evidence from a cohort study in rural Uganda. AIDS Behav..

[7] Stangl AL, Pliakas T, Mainga T, Steinhaus M, Mubekapi-Musadaidzwa C, Viljoen L (2021). The effect of universal testing and treatment on HIV stigma in 21 communities in Zambia and South Africa. AIDS..

[8] Turan B, Hatcher AM, Weiser SD, Johnson MO, Rice WS, Turan JM (2017). Framing mechanisms linking HIV-related stigma, adherence to treatment, and health outcomes. Am J Public Health..

[9] Nyblade L, Srinivasan K, Mazur A, Raj T, Patil DS, Devadass D (2018). HIV stigma reduction for health facility staff: development of a blended- learning intervetion. Front Public Health..

[10] Ikeda DJ, Nyblade L, Srithanaviboonchai K, Agins BD (2019). A quality improvement approach to the reduction of HIV-related stigma and discrimination in healthcare settings. BMJ Glob Health..

[11] Gottert A, McClair TL, Pulerwitz J, Friedland BA (2020). the PLHIV Stigma Index 2.0 Study Group in Cambodia, the Dominican Republic and Uganda. What shapes resilience among people living with HIV? A multi-country analysis of data from the PLHIV Stigma Index 2.0. AIDS..

[12] Stangl AL, Earnshaw VA, Logie CH, van Brakel W, Simbayi LC, Barre I (2019). The health stigma and discrimination framework: a global, crosscutting framework to inform research, intervention development, and policy on health-related stigmas. BMC Med..

[13] Goffman E (1963). Stigma: Notes on the management of spoiled identity.

[14] Link BG, Phelan JC (2001). Conceptualizing stigma. Annu Rev Sociol..

[15] Lubkin IM, Larsen PD. Chronic illness: impact and intervention. 8^th^ edition. Burlington: Jones & Bartlett Learning; 2013.

[16] Deacon H, Stephney I, Prosalendis S (2005). Understanding HIV/AIDS stigma: a theoretical and methodological analysis.

[17] Thornicroft G, Rose D, Kassam A, Sartorius N (2007). Stigma: ignorance, prejudice or discrimination?. Br J Psychiatry..

[18] UNAIDS. Fact sheet: stigma and discrimination. Joint United Nations Programme on HIV/AIDS. 2003. https://data.unaids.org/publications/fact-sheets03/fs_stigma_discrimination_en.pdf. Accessed 24 Mar 2021.

[19] Thapa S, Hannes K, Cargo M, Buve A, Aro AR, Mathei C (2017). Building a conceptual framework to study the effect of HIV stigma-reduction intervention strategies on HIV test uptake: a scoping review. J Assoc Nurses AIDS Care..

[20] Woodford MR, Chakrapani V, Newman PA, Shunmugam M (2016). Barriers and facilitators to voluntary HIV testing uptake among communities at high risk of HIV exposure in Chennai, India. Global public health..

[21] Gesesew HA, Tesfay Gebremedhin A, Demissie TD (2017). Significant association between perceived HIV related stigma and late presentation for HIV/AIDS care in low and middle-income countries: a systematic review and meta-analysis. PLoS One..

[22] Katz IT, Ryu AE, Onuegbu AG, Psaros C, Weiser SD, Bangsberg DR (2013). Impact of HIV-related stigma on treatment adherence: systematic review and meta-synthesis. J Int AIDS Soc..

[23] Demeke HB (2013). Relationships between HIV-related stigma, coping, social support and health-related quality of life in people living with HIV/AIDS [dissertation].

[24] Holzemer WL, Human S, Arudo J, Rosa ME, Hamilton MJ, Corless I (2009). Exploring HIV stigma and quality of life for persons living with HIV infection. J Assoc Nurses AIDS Care..

[25] Danforth K, Granich R, Wiedeman D, Baxi S, Padian N. Global mortality and morbidity of HIV/AIDS. In: Holmes KK, Bertozzi S, Bloom BR, Jha P, editors. Major infectious diseases. 3rd ed. Washington, DC: The International Bank for Reconstruction and Development / The World Bank; 2017;2:29-44. 30212096

[26] UNAIDS. Global partnership for action to eliminate all forms of HIV-related stigma and discrimination. 2018. https://www.unaids.org/en/resources/documents/2018/global partnership-hiv-stigma-discrimination. Accessed 23 Mar 2021.

[27] UNAIDS. UNAIDS data 2020. 2020. https://www.unaids.org/sites/default/files/media_asset/2020_aids-data-book_en.pdf. Accessed 10 Apr 2021.

[28] Genberg BL, Kawichai S, Chingono A, Sendah M, Chariyalertsak S, Konda KA, Celentano DD (2008). Assessing HIV/AIDS stigma and discrimination in developing countries. AIDS and Behavior..

[29] Geter A, Herron AR, Sutton MY (2018). HIV-related stigma by healthcare providers in the United States: a systematic review. AIDS Patient Care STDS..

[30] Kredo T, Ford N, Adeniyi FB, Garner P. Decentralising HIV treatment in lower- and middle-income countries. Cochrane Database Syst Rev. 2013;(6):CD009987. 10.1002/14651858.CD009987.pub2. 10.1002/14651858.CD009987.pub2 PMC10009870 23807693

[31] Mazzola LT, Pérez-Casas C. HIV/AIDS diagnostics technology landscape 5th edition. 2015. http://www.unitaid.org/assets/UNITAID_HIV_Nov_2015_Dx_Landscape-1.pdf. Accessed 10 Apr 2021.

[32] Onwujekwe O, Chikezie I, Mbachu C, Chiegil R, Torpey K, Uzochukwu B (2016). Investigating client perception and attitude to decentralization of HIV/AIDS treatment services to primary health centres in three Nigerian states. Health Expect..

[33] Zhang X, Miège P, Zhang Y. Decentralization of the provision of health services to people living with HIV/AIDS in rural China: the case of three counties. Health Res Policy Syst. 2011. 10.1186/1478-4505-9-9. 10.1186/1478-4505-9-9 PMC3045992 21310093

[34] Bilinski A, Birru E, Peckarsky M, Herce M, Kalanga N, Neumann C (2017). Distance to care, enrollment and loss to follow-up of HIV patients during decentralization of antiretroviral therapy in Neno district, Malawi: a retrospective cohort study. PLoS ONE..

[35] Kolawole GO, Gilbert HN, Dadem NY, Genberg BL, Agaba PA, Okonkwo P (2017). Patient experiences of decentralized HIV treatment and care in Plateau State, North Central Nigeria: a qualitative study. AIDS Res Treat..

[36] Abongomera G, Chiwaula L, Revill P, Mabugu T, Tumwesige E, Nkhata M (2018). Patient-level benefits associated with decentralization of antiretroviral therapy services to primary health facilities in Malawi and Uganda. Int Health..

[37] Mdege ND, Chindove S, Ali S (2013). The effectiveness and cost implications of task-shifting in the delivery of antiretroviral therapy to HIV-infected patients: a systematic review. Health Policy Plan..

[38] Kredo T, Adeniyi FB, Bateganya M, Pienaar ED. Task shifting from doctors to non-doctors for initiation and maintenance of antiretroviral therapy. Cochrane Database Syst Rev. 2014;(7):CD007331. 10.1002/14651858.CD007331.pub3. 10.1002/14651858.CD007331.pub3 PMC11214583 24980859

[39] Suthar AB, Rutherford GW, Horvath T, Doherty M, Negussie EK (2014). Improving antiretroviral scale-up and effectiveness through service integration and decentralization. AIDS..

[40] Vorasane S, Jimba M, Kikuchi K, Yasuoka J, Nanishi K, Durham J, Sychareun V (2017). An investigation of stigmatizing attitudes towards people living with HIV/AIDS by doctors and nurses in Vientiane. Lao PDR. BMC Health Serv Res..

[41] Dawson-Amoah CG. Determinants of HIV stigma among healthcare workers in Ghana [dissertation]. Walden University; 2015. https://scholarworks.waldenu.edu/cgi/viewcontent.cgi?article=2530&context=dissertations. Accessed 13 Apr 2021.

[42] Waluyo A, Culbert GJ, Levy J, Norr KF (2015). Understanding HIV-related stigma among Indonesian nurses. J Assoc Nurses AIDS Care..

[43] Stringer KL, Turan B, McCormick L, Durojaiye M, Nyblade L, Kempf MC (2016). HIV-related stigma among healthcare providers in the deep south. AIDS Behav..

[44] UNAIDS. Getting to zero: 2011-2015 strategy. 2010. https://unaids-test.unaids.org/sites/default/files/unaids/contentassets/documents/unaidspublication/2010/20101221_JC2034E_UNAIDS-Strategy_en.pdf. Accessed 10 Apr 2021.

[45] UNAIDS. Implementation of the HIV prevention 2020 road map, Nov 2020. 2020. https://www.unaids.org/sites/default/files/media_asset/fourth-annual-progress-report-global-hiv-prevention-coalition_en.pdf. Accessed 10 Apr 2021.

[46] Ministry of Health, Republic of Indonesia. Report on the Development of HIV AIDS and Sexually Transmitted Disease, Fist Quarter of 2021, Report No: PM.02.02/III/137/2021. Jakarta: Ministry of Health of Republic of Indonesia.

[47] National AIDS Commission, the Ministry of Health of Republic of Indonesia. Roadmap to reduce HIV-related morbidity and mortality and maximize the prevention benefits of scaling-up access to ARVs: rapid scaling-up of HIV testing and treatment in high burden districts 2013-2015. Jakarta: National AIDS Commission and the Ministry of Health of Republic of Indonesia.

[48] Ministry of Health, Republic of Indonesia. Indonesia HIV National Program and HIV in Elderly Situation: Facilities for HIV Testing and Treatment. Presented at: University Center of Excellence- AIDS Research Center International Symposium 2021. Ministry of Health of Republic of Indonesia .

[49] Sianturi EI, Perwitasari DA, Islam MA, Taxis K (2019). The association between ethnicity, stigma, beliefs about medicines and adherence in people living with HIV in a rural area in Indonesia. BMC Public Health..

[50] Wardojo SSI, Huang Y-L, Chuang K-Y (2021). Determinants of the quality of life amongst HIV clinic attendees in Malang, Indonesia. BMC Public Health..

[51] Harapan H, Khalilullah SA, Anwar S, Zia M, Novianty F, Putra RP (2015). Discriminatory attitudes toward people living with HIV among health care workers in Aceh, Indonesia: a vista from a very low HIV caseload region. Clin Epidemiol Glob Health..

[52] Wolitski RJ, Pals SL, Kidder DP, Courtenay-Quirk C, Holtgrave DR (2009). The effects of HIV stigma on health, disclosure of HIV status, and risk behavior of homeless and unstably housed persons living with HIV. AIDS and Behavior..

[53] Statistics Indonesia. [New Methods] Human Development Index 2020-2021. 2022. https://www.bps.go.id/indicator/26/413/1/-metode-baru-indeks-pembangunan-manusia.html. Accessed 13 Feb 2022.

[54] Central Bureau of Statistics. Population size by age group and sex in Gunungkidul district. 2017. https://gunungkidulkab.bps.go.id/statictable/2017/08/03/60/jumlah-penduduk-menurut-golongan-umur-dan-jenis-kelamin-di-kabupaten-gunungkidul-2016.html. Accessed 12 Apr 2021.

[55] Gunungkidul Health Office. Health profile of gunungkidul district 2015. Gunungkidul: Gunungkidul Health Office; 2016.

[56] Pratikno, H. Stigma and discrimination against PLHIV (people living with HIV AIDS) in Bengkalis District, Riau Province [thesis]. Gadjah Mada University; 2008

[57] The Health Policy Project. Measuring HIV stigma and discrimination among health facility staff: standardized brief questionnaire. 2013. https://www.healthpolicyproject.com/pubs/49_StandardizedBriefQuestionnaireMeasuringSD.pdf. Accessed 10 Mar 2021.

[58] Risal A, Irwan AM, Sjattar EL (2018). Stigma towards people living with HIV/AIDS among counseling officers in South Sulawesi. Indonesia. Belitung Nurs J..

[59] The Ministry of Health Republic of Indonesia. Basic Health Research 2010: a report. Jakarta: Health Research and Development Agency; 2010.

[60] Health Policy Initiative, Task Order 1. Measuring the degree of HIV-related stigma and discrimination in health facilities and providers: working report; Washington, DC: Futures; 2010.

[61] Williams, R. Analyzing proportions: fractional response and zero one inflated beta models*.* 2019. https://www3.nd.edu/~rwilliam/stats3/fractionalresponsemodels.pdf. Accessed 26 Feb 2021.

[62] Feyissa GT, Abebe L, Girma E, Woldie M (2012). Stigma and discrimination against people living with HIV by healthcare providers, Southwest Ethiopia. BMC Public Health..

[63] Li L, Wu Z, Wu S, Zhaoc Y, Jia M, Yan Z (2007). HIV-related stigma in health care settings: a survey of service providers in China. AIDS Patient Care STDs..

[64] Yang H, Li X, Stanton B, Fang X, Lin D, Naar-King S (2006). HIV-related knowledge, stigma, and willingness to disclose: a mediation analysis. AIDS care..

[65] Mutevedzi PC, Lessells RJ, Heller T, Bärnighausen T, Cooke GS, Newell ML. Scale-up of a decentralized HIV treatment programme in rural KwaZulu-Natal, South Africa: does rapid expansion affect patient outcomes? Bull World Health Organ. 2010;88(8):593-600. https://apps.who.int/iris/handle/10665/270740. Accessed 16 May 2021. 10.2471/BLT.09.069419 PMC2908968 20680124

[66] Reidy WJ, Sheriff M, Wang C, Hawken M, Koech E, Elul B (2014). Decentralization of HIV care and treatment services in Central Province, Kenya. J Acquir Immune Defic Syndr..

[67] Edwards N, Kaseje D, Kahwa E, Klopper HC, Mill J, Webber J (2015). The impact of leadership hubs on the uptake of evidence-informed nursing practices and workplace policies for HIV care: a quasi-experimental study in Jamaica, Kenya, Uganda and South Africa. Implement Sc..

[68] Airhihenbuwa CO, Ford CL, Iwelunmor JI (2014). Why culture matters in health interventions: lessons from HIV/AIDS stigma and NCDs. Health Educ Behav..

[69] Djalil MA, Lubis FAR (2020). The effect of work rotation and work culture on work satisfaction and work skill and its impact on employee performance of Dr. Zainoel Abidin Regency Hospital, Banda Aceh, Indonesia. East African Scholars J Econ Bus Manag..

