# Supplementary material for: HIV-related stigma and discrimination among health care workers during early program decentralization in rural district Gunungkidul, Indonesia: a cross-sectional study
Source: BMC Health Serv Res. 2022 Mar 17;22:356. doi: 10.1186/s12913-022-07751-7 (PMC8932246; doi:10.1186/s12913-022-07751-7)
Supplement: Supplementary file 1 — Additional file 1. [file 12913_2022_7751_MOESM1_ESM.docx]

**Table A1.** SAD prevalence and PHC sampling rates

| PHC | Sampling rate, |  | SAD outcome, *d*/*n* (%) | | | |
| --- | --- | --- | --- | --- | --- | --- |
| site | *d*/*n* (%) |  | Fear of | Perceived image | Avoidance of | Discriminatory |
|  |  |  | transmission^a^ | of PLHIV^a^ | service duties^a^ | practice^a^ |
| 1 | 11/23 (47.8%) |  | 7/9 (77.8%)^b^ | 6/11 (54.5%) | 9/11 (81.8%) | 7/7 (100.0%)^b^ |
| 2 | 6/15 (40.0%) |  | 3/6 (50.0%) | 4/6 (66.7%) | 5/6 (83.3%) | 2/5 (40.0%)^b^ |
| 3 | 13/27 (48.1%) |  | 7/11 (63.6%)^b^ | 8/13 (61.5%) | 10/13 (76.9%) | 7/10 (70.0%)^b^ |
| 4 | 6/17 (35.3%) |  | 2/5 (40.0%)^b^ | 6/6 (100.0%) | 2/6 (33.3%) | 4/5 (80.0%)^b^ |
| 5 | 9/21 (42.9%) |  | 4/8 (50.0%)^b^ | 5/9 (55.6%) | 9/9 (100.0%) | 6/6 (100.0%)^b^ |
| 6 | 11/17 (64.7%) |  | 9/10 (90.0%)^b^ | 8/11 (72.7%) | 6/11 (54.5%) | 9/10 (90.0%)^b^ |
| 7 | 10/17 (58.8%) |  | 7/9 (77.8%)^b^ | 9/10 (90.0%) | 5/10 (50.0%) | 5/6 (83.3%)^b^ |
| 8 | 7/22 (31.8%) |  | 5/6 (83.3%)^b^ | 4/7 (57.1%) | 3/7 (42.9%) | 1/3 (33.3%)^b^ |
| 9 | 6/20 (30.0%) |  | 6/6 (100.0%) | 5/6 (83.3%) | 3/6 (50.0%) | 6/6 (100.0%) |
| 10 | 8/18 (44.4%) |  | 6/8 (75.0%) | 8/8 (100.0%) | 3/8 (37.5%) | 7/7 (100.0%)^b^ |
| 11 | 9/24 (37.5%) |  | 6/8 (75.0%)^b^ | 8/9 (88.9%) | 4/9 (44.4%) | 5/5 (100.0%)^b^ |
| 12 | 10/20 (50.0%) |  | 5/7 (71.4%)^b^ | 9/10 (90.0%) | 6/10 (60%) | 8/9 (88.9%)^b^ |
| 13 | 12/24 (50.0%) |  | 7/10 (70%)^b^ | 9/12 (75.0%) | 10/12 (83.3%) | 10/11 (90.9%)^b^ |
|  |  |  |  |  |  |  |
|  | *p*-value^c^ |  | 0.685 | 0.958 | 0.323 | 0.308 |
|  |  |  |  |  |  |  |
| *d* = number sampled or with outcome; *n* = number of available PHC staff or sampled; PHC = prima- | | | | | | |
| ry health center; SAD = stigma and discrimination. | | | | | | |
| ^a^ = Fear of HIV transmission: how worry staff are with the prospect of HIV transmission when | | | | | | |
| providing care to people living with HIV (PLHIV); perceived image of PLHIV: unfounded | | | | | | |
| beliefs, presumptions of negative behaviors of PLHIV; avoidance of service duties: omission, | | | | | | |
| neglect to provide services for HIV key populations if such an option becomes feasible; | | | | | | |
| discriminatory practice: unnecessary preventive measures taken when caring for PLHIV. | | | | | | |
| ^b^ = *n* denotes the number of eligible responses in the analysis of SAD outcomes. | | | | | | |
| ^c^ = *p*-value of the association between sampling rates and SAD outcomes, obtained from binomial re- | | | | | | |
| gression with a logit link function. | | | | | | |
